# Novel eRF3a degrader enhances gentamicin-induced premature termination codon readthrough in epidermolysis bullosa

**DOI:** 10.1016/j.omtn.2025.102741

**Published:** 2025-10-13

**Authors:** Kathleen L. Miao, Brandon Levian, Yingping Hou, Ryan Huynh, Kate Zheng, Mei Chen

**Affiliations:** 1 Department of Dermatology, The Keck School of Medicine, University of Southern California, Los Angeles, CA, USA

**Keywords:** MT: Delivery Strategies, epidermolysis bullosa, extracellular matrix, genetic diseases, readthrough therapy, skin

## Abstract

Recessive dystrophic epidermolysis bullosa (RDEB) and junctional epidermolysis bullosa (JEB) are severe blistering skin disorders caused by mutations in genes encoding type VII collagen (*COL7A1*) and laminin 332 (*LAMA3*, *LAMB3*, or *LAMC2*), respectively. In RDEB, 25% of patients carry nonsense mutations that result in premature termination codons (PTCs), while in JEB, the majority of mutations in *LAMB3* are nonsense mutations (80%). CC-90009, an eRF3a degrader, is effective in inducing PTC readthrough in various *in vitro* models of diseases caused by nonsense mutations. This study evaluated CC-90009’s ability, in combination with gentamicin, to suppress PTCs and promote the expression of type VII collagen (C7) in primary RDEB keratinocytes and fibroblasts, as well as laminin 332 in primary JEB keratinocytes with nonsense mutations. While CC-90009 alone demonstrated limited efficacy, its combination with low-dose gentamicin led to a dose-dependent increase in C7 and laminin β3 production, surpassing the effects of high-dose gentamicin alone. Furthermore, CC-90009/gentamicin reversed the hypermotility and poor substratum attachment characteristic of EB cells. Finally, C7 and laminin 332 induced by CC-90009/gentamicin localized to the dermal-epidermal junction in RDEB and JEB skin equivalents. Therefore, CC-90009/gentamicin may present a novel and safe treatment option for RDEB, JEB, and other inherited skin diseases arising from nonsense mutations.

## Graphical abstract

**Figure:**
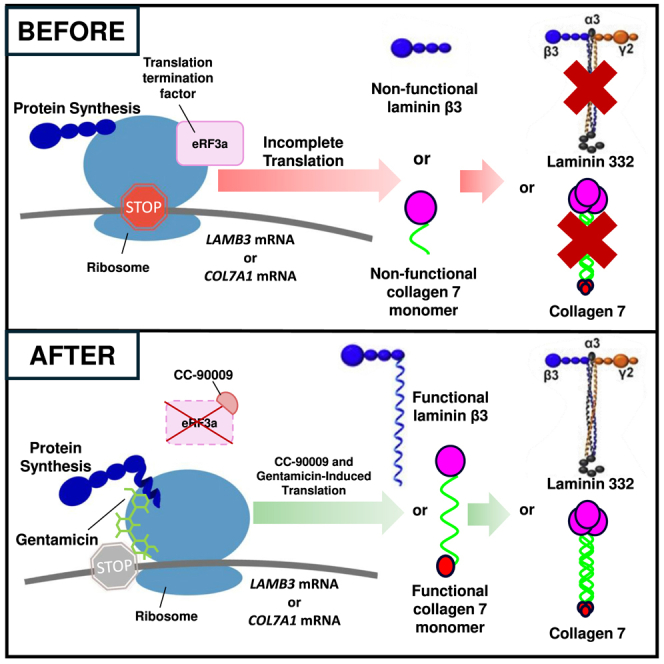

## Teaser

Miao and colleagues investigated CC-90009, an eRF3a degrader, to enhance gentamicin-induced premature termination codon readthrough, restoring functional type VII collagen and laminin 332 in RDEB and JEB cells. Combined with low-dose gentamicin, CC-90009 may offer a novel, safer therapy for RDEB, JEB, and other inherited skin disorders caused by nonsense mutations.

## Introduction

Epidermolysis bullosa (EB) encompasses a group of skin disorders marked by the formation of severe blisters and scarring.^1^ Recessive dystrophic epidermolysis bullosa (RDEB), a member of the EB family inherited in an autosomal recessive manner, arises from mutations in the *COL7A1* gene, leading to a deficiency or absence of type VII collagen (C7).^2^ In normal human skin, fibroblasts and keratinocytes synthesize C7, which form anti-parallel dimers that aggregate into anchoring fibrils (AFs). These AFs maintain dermal-epidermal junction (DEJ) integrity by securing the dermis to the epidermis.^3^ For individuals with RDEB, poor dermal-epidermal adherence causes complications such as syndactyly, esophageal stenosis, ankyloglossia, microstomia, and severe joint contractures.^4^^,^^5^ Frequent cycles of skin injury and repair commonly lead to the emergence of lethal and aggressive squamous cell carcinomas, often culminating in mortality by middle adulthood.^6^

While therapeutic approaches such as protein therapy, cell therapy, and recently Food and Drug Administration (FDA)-approved localized gene therapies—including Vyjuvek (beremagene geperpavec, a herpes simplex virus 1-based gene therapy) and Zevaskyn (prademagene zamikerace, a gene-corrected keratinocyte autograft transplantation)—as well as Filsuvez, a birch bark extract, now exist, EB care currently remains largely focused on wound prevention and infection management.^7^^,^^8^^,^^9^^,^^10^

Junctional epidermolysis bullosa (JEB), another EB subtype, induces widespread blistering, frequently resulting in persistent infections, feeding difficulties, and treatment-resistant anemia.^11^ Mutations in *LAMA3*, *LAMB3*, and *LAMC2* genes lead to reduced or absent laminin 332, crucial for the formation of anchoring filaments and DEJ adherence.^12^^,^^13^ Despite interventions like protein replacement therapy, gene therapy, and bone marrow transplantation, most JEB patients do not survive beyond infancy.^14^^,^^15^^,^^16^^,^^17^^,^^18^

Nonsense mutations give rise to premature termination codons (PTCs), resulting in unstable mRNA transcripts that are either broken down or translated into a shortened and non-functional polypeptide.^19^ Approximately 10%–25% of RDEB cases and 95% of JEB-associated *LAMB3* mutations involve nonsense mutations, with *LAMB3* mutations accounting for over 80% of severe JEB cases.^11^^,^^20^ Nonsense-mediated readthrough therapy (NMRT) suppresses PTCs, thereby enabling the generation of full-length protein products.^21^ Aminoglycosides such as gentamicin have previously shown efficacy in inducing NMRT in different genetic disorders attributed to nonsense mutations. In prior studies, we have established that gentamicin possesses the capability to induce PTC readthrough, resulting in the synthesis of complete C7 or laminin 332 in cultured cells of individuals with RDEB and JEB, respectively.^22^^,^^23^ Subsequent investigations demonstrated that topical, intradermal, intramuscular, and intravenous administration of gentamicin-induced PTC readthrough, increasing the production of C7 and laminin 332 in patients with RDEB and JEB and leading to enhanced wound healing.^24^

Although aminoglycosides, including gentamicin, have shown the capacity to induce PTC readthrough and benefit individuals with EB, the extended clinical use of aminoglycosides is constrained by concerns for nephrotoxicity and ototoxicity.^25^^,^^26^ Therefore, recent research has focused on identifying compounds that can synergistically enhance the readthrough activity of aminoglycosides while minimizing their associated toxicity. One such compound is CC-90009, an eukaryotic release factor 3a (eRF3a) degrader currently in phase 1 trials for acute myeloid leukemia.^27^^,^^28^^,^^29^^,^^30^^,^^31^ CC-90009 has shown efficacy in inducing PTC readthrough in various *in vitro* disease models caused by nonsense mutations, such as mucopolysaccharidosis type I (Hurler syndrome), Duchenne muscular dystrophy, cystic fibrosis, and retinoblastoma.^32^^,^^33^^,^^34^ Furthermore, CC-90009 has exhibited its capacity to act synergistically with various compounds including aminoglycosides to dramatically enhance PTC readthrough capability including JEB cells harboring mutations in the gene coding for type XVII collagen (*COL17A1*).^34^^,^^35^^,^^36^ However, to date, a combination therapy utilizing both CC-90009 and gentamicin has not been tested in RDEB and JEB cells harboring nonsense mutations in C7 and laminin 332, respectively.

In this study, we aimed to assess the feasibility of using a combination of CC-90009 and low-dose gentamicin to induce PTC readthrough and restore C7 and laminin 332 in RDEB and JEB cells with nonsense mutations. We showed that CC-90009/gentamicin combination significantly increased C7 and laminin β3 synthesis, outperforming high-dose gentamicin alone. Additionally, the combination improved EB cell adhesion and motility, with induced C7 and laminin 332 localizing to the DEJ in skin equivalents (SEs).

## Results

### CC-90009 and gentamicin combination therapy induces a synergistic production of full-length C7 in RDEB fibroblasts

**Figure 1:**
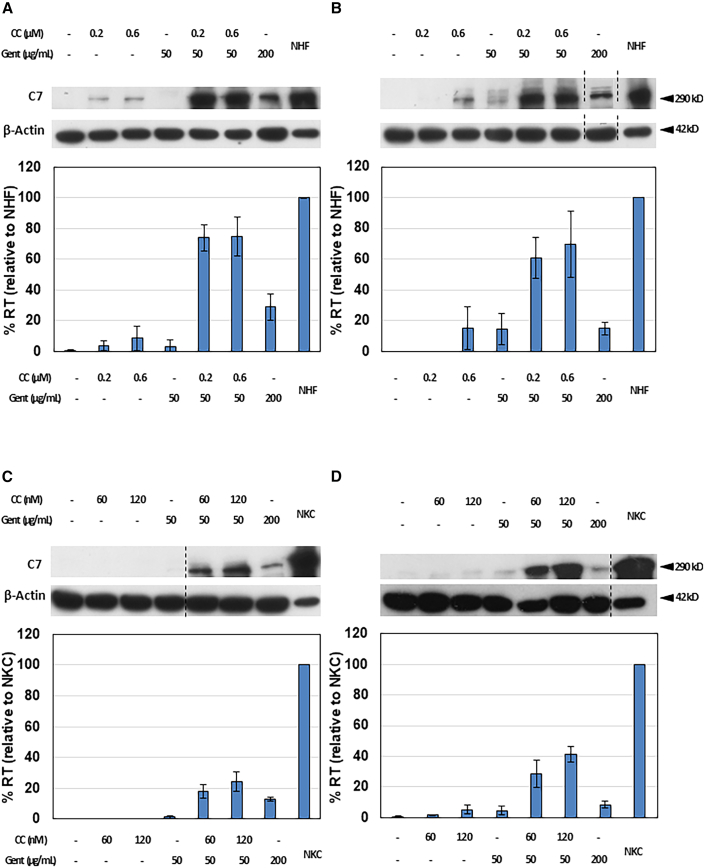
CC-90009 enhances gentamicin-induced full-length C7 production in RDEB fibroblasts and keratinocytes RDEB fibroblasts, denoted as RDEB1 (A) and RDEB2 (B), and RDEB keratinocytes, identified as RDEB1 (C) and RDEB2 (D), were treated with increasing concentrations of CC-90009 (CC) and gentamicin (Gent) as indicated, for 48 h. Cell lysates were prepared and then subjected to 4%–12% SDS-PAGE, followed by immunoblot analysis with a rabbit polyclonal antibody to the NC1 domain of C7 or anti-β-actin (loading control) antibody. ImageJ analysis of C7 expression normalized with β-actin is shown below the respective blots. The results are displayed as compared to normal human fibroblasts (NHF) in RDEB fibroblasts and compared to normal human keratinocytes (NKC) in RDEB keratinocytes. Dashed black lines indicate where the gel is cropped from the same blot. Error bars, SE of three different experiments. RT, readthrough.

In our exploration of CC-90009’s viability as a prospective treatment for RDEB caused by nonsense mutations, we conducted experiments involving various concentrations of CC-90009 and/or gentamicin. The observed effects were then compared to those of gentamicin and normal human fibroblasts (NHFs) in primary RDEB fibroblast cells originating from two individuals with RDEB carrying nonsense mutations (RDEB1 and RDEB2). RDEB1 cells are homozygous for R578X mutations, whereas RDEB2 cells are heterozygous for R613X and R1683X mutations. RDEB fibroblasts were exposed to escalating concentrations of CC-90009 and/or gentamicin, followed by subsequent immunoblot analysis of cell lysates. Treatment with the optimal concentration of CC-90009 (0.6 μM) as a monotherapy only resulted in a full-length C7 production that was 8.4% and 15.2% of that seen in NHFs for RDEB1 and RDEB2, respectively (Figures 1A and 1B). Subsequently, the optimal concentration for the combined treatment of CC-90009 and low-dose gentamicin was established for RDEB1 and RDEB2, which was determined to be 0.6 μM CC-90009 plus 50 μg/mL gentamicin. Under the optimal concentrations of CC-90009 and low-dose gentamicin combination therapy, RDEB1 cells exhibited a C7 expression level that was 74.6% in comparison to NHFs and 2.6-fold greater than that of high-dose gentamicin alone (200 μg/mL). Similarly, RDEB2 cells exhibited a C7 expression level that was 69.4% of that seen in NHFs and 4.7-fold greater than that of high-dose gentamicin alone (200 μg/mL) (Figures 1A and 1B). Untreated parent cells exhibited negligible or no C7 expression, and no evidence of cellular cytotoxicity was observed across the range of CC-90009 and/or gentamicin concentrations tested earlier (Figure S1). It is important to emphasize that combination therapy involving CC-90009 and gentamicin yielded synergistic outcomes, as opposed to being merely additive. These results suggest that combination therapy with CC-90009 and low-dose gentamicin has the capability to induce PTC readthrough and promote a synergistic production of full-length C7 in RDEB fibroblasts.

### CC-90009 and gentamicin combination therapy induces a synergistic production of full-length C7 in RDEB keratinocytes

Considering the joint involvement of epidermal fibroblasts and keratinocytes in C7 production, we further explored whether the combination therapy of CC-90009 and gentamicin could induce comparable PTC readthrough and full-length C7 synthesis in RDEB keratinocytes. To determine this, we utilized primary keratinocytes from the same two RDEB patients with nonsense mutations (RDEB1 and RDEB2). Similar to RDEB fibroblasts, RDEB keratinocytes were exposed to escalating concentrations of CC-90009 and/or gentamicin, followed by subsequent immunoblot analysis of cell lysates. CC-90009 monotherapy demonstrated limited effectiveness for both cell lines (Figures 1C and 1D). Nevertheless, at the ideal concentration for the combination therapy of CC-90009 and gentamicin (120 nM CC-90009 plus 50 μg/mL gentamicin), the C7 expression level in RDEB1 keratinocytes was 24.2% in comparison to normal human keratinocytes (NKCs) and 1.9-fold greater than that of high-dose gentamicin (200 μg/mL) alone (Figure 1C). Furthermore, at the ideal concentration for the combination therapy of CC-90009 and gentamicin for RDEB2 keratinocytes (120 nM CC-90009 plus 50 μg/mL gentamicin), the C7 expression level in RDEB2 keratinocytes was 5.0 times higher than that achieved with high-dose gentamicin alone (200 μg/mL) and 41.4% relative to NKCs (Figure 1D). Cell media from RDEB keratinocytes were harvested, concentrated, and then subjected to immunoblot analyses. The results show that C7 induced by CC-90009/low-dose gentamicin or high-dose gentamicin alone was secreted into the medium as well (Figure S2). No evidence of cellular cytotoxicity was observed across the range of CC-90009 and/or gentamicin concentrations tested earlier (Figure S3). Consistent with the findings in RDEB fibroblasts, it is important to emphasize that the combination therapy of CC-90009 and gentamicin exhibited synergistic effects in all RDEB keratinocytes, rather than simply cumulative results. These results indicate that combination therapy with CC-90009 and gentamicin has the capability to induce PTC readthrough and promote a synergistic production of full-length C7 in both RDEB fibroblasts and keratinocytes.

### Production of full-length C7 in RDEB fibroblasts and keratinocytes increases with multiple doses of CC-90009 and gentamicin combination therapy

**Figure 2:**
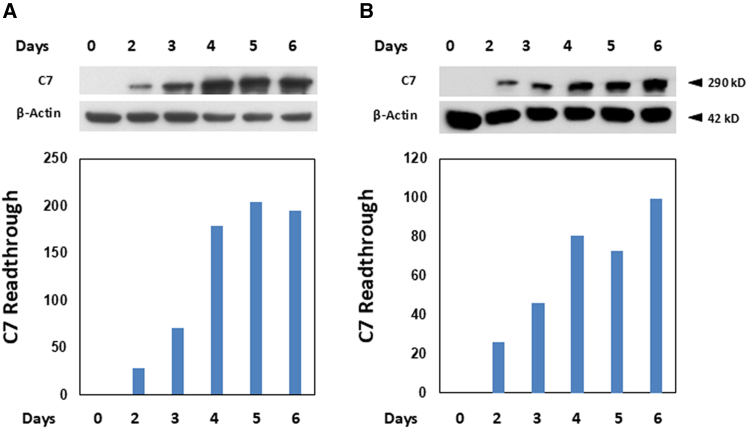
CC-90009 and gentamicin-induced production of full-length C7 increased with continued dosing RDEB1 fibroblasts (A) and RDEB1 keratinocytes (B) were incubated with growth media in the absence of CC-90009/gentamicin or were given consecutive daily treatments of CC-90009/gentamicin for up to 6 days. RDEB1 fibroblasts were treated with 100 nM CC-90009 and 50 μg/mL gentamicin, while RDEB1 keratinocytes were treated with 30 nM CC-90009 and 50 μg/mL gentamicin. Cell lysates were prepared and then subjected to 4%–12% SDS-PAGE, followed by immunoblot analysis with a rabbit polyclonal antibody to the NC1 domain of C7 or anti-β-actin (loading control) antibody. The results are displayed as a fraction of the level of C7 obtained from the 6th dose of CC-90009/gentamicin. Please note that CC-90009/gentamicin-induced full-length C7 expression that trended positively with daily treatment.

In the clinical setting, patients typically receive successive doses of a therapeutic regimen. Consequently, we aimed to more accurately mimic clinical practice by investigating the impact of extended dosing with CC-90009 and gentamicin combination therapy on the production of C7 in RDEB fibroblasts and keratinocytes. RDEB1 fibroblasts and keratinocytes were cultured in growth medium supplemented daily with 100 nM CC-90009 plus 50 μg/mL gentamicin (fibroblasts) or 30 nM CC-90009 plus 50 μg/mL gentamicin (keratinocytes) for 6 consecutive days. RDEB1 fibroblasts and keratinocytes demonstrated an increasing trend in C7 production with each subsequent administration of CC-90009 and gentamicin combination therapy (Figures 2A and 2B). A parallel experiment using higher concentrations—0.6 μM CC-90009 in fibroblasts and 120 nM in keratinocytes—administered over 5 days similarly demonstrated a dose-dependent increase in C7 levels (Figure S4). Thus, these results demonstrate that successive administrations of CC-90009 and gentamicin combination therapy lead to increasing levels of PTC readthrough and, consequently, elevated C7 production in RDEB fibroblasts and keratinocytes.

### CC-90009 and gentamicin combination therapy induces a synergistic production of laminin 332 in JEB keratinocytes

**Figure 3:**
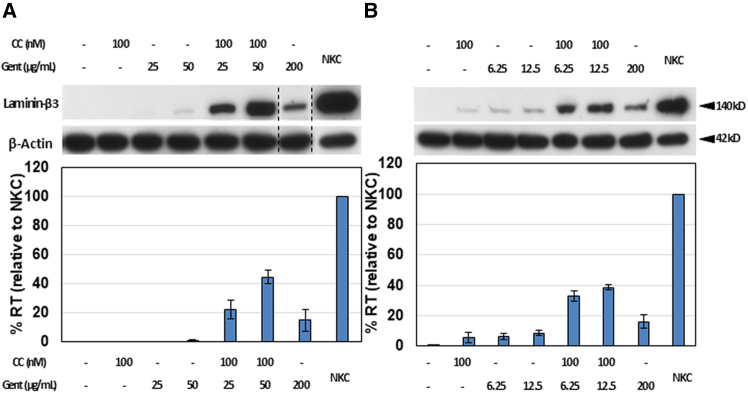
CC-90009 enhanced gentamicin-mediated induction of full-length laminin β3 in JEB keratinocytes JEB1 (A) and JEB2 (B) primary keratinocytes were treated with increasing concentrations of CC-90009 (CC) and gentamicin (Gent) as indicated, for 48 h. Cell lysates were prepared and then subjected to 4%–12% SDS-PAGE, followed by immunoblot analysis with a monoclonal anti-laminin β3 antibody or an anti-β-actin (loading control) antibody. ImageJ analysis of laminin β3 expression normalized with β-actin is shown below the respective blots. The results are displayed as compared to normal human keratinocytes (NKCs). Dashed black lines indicate where the gel is cropped from the same blot. Error bars, SE of three different experiments. RT, readthrough.

Given that a predominant portion of JEB-causing mutations are characterized as nonsense mutations, our objective was to assess the efficacy of CC-90009/gentamicin combination therapy in JEB keratinocytes. We utilized JEB keratinocytes derived from two individuals with JEB carrying nonsense mutations in *LAMB3* (JEB1 and JEB2). JEB1 cells are heterozygous for R42X and R635X mutations, and JEB2 cells are heterozygous for C325X/c.629-12T>A mutations. JEB keratinocytes were exposed to escalating concentrations of CC-90009 and/or gentamicin, followed by subsequent immunoblot analysis of cell lysates. CC-90009 monotherapy demonstrated minimal effectiveness for JEB1 and JEB2 cells. As shown in Figures 3A and 3B, CC-90009 or low-dose gentamicin alone induced minimal readthrough and laminin β3 expression. However, at the optimal concentration for the combination therapy of CC-90009 and gentamicin for JEB1 (100 nM CC-90009 plus 50 μg/mL gentamicin) and JEB2 (100 nM CC-90009 plus 12.5 μg/mL gentamicin) cells, the laminin β3 expression level in JEB1 cells was 3.0 times greater than that achieved with high-dose gentamicin alone (200 μg/mL) and 44.7% of that seen in NKCs (Figure 3A). Laminin β3 expression in JEB2 cells was 2.4 times greater than that achieved with high-dose gentamicin alone (200 μg/mL) and 38.7% of that seen in NKCs (Figure 3B). No evidence of cellular cytotoxicity was observed across the range of CC-90009 and/or gentamicin concentrations tested earlier (Figure S5). The limited efficacy of CC-90009 monotherapy in JEB keratinocytes closely mirrored the outcomes observed in RDEB keratinocytes. However, consistent with observations in RDEB fibroblasts and keratinocytes, the combination therapy of CC-90009 and gentamicin demonstrated synergistic effects in JEB keratinocytes, rather than merely producing combined effects. These findings suggest that the combined therapy involving CC-90009 and gentamicin has the potential to induce PTC readthrough and foster a synergistic production of full-length laminin β3 in JEB keratinocytes.

### CC-90009 and gentamicin combination therapy reverses the hypermotility characteristic of RDEB fibroblasts and keratinocytes

**Figure 4:**
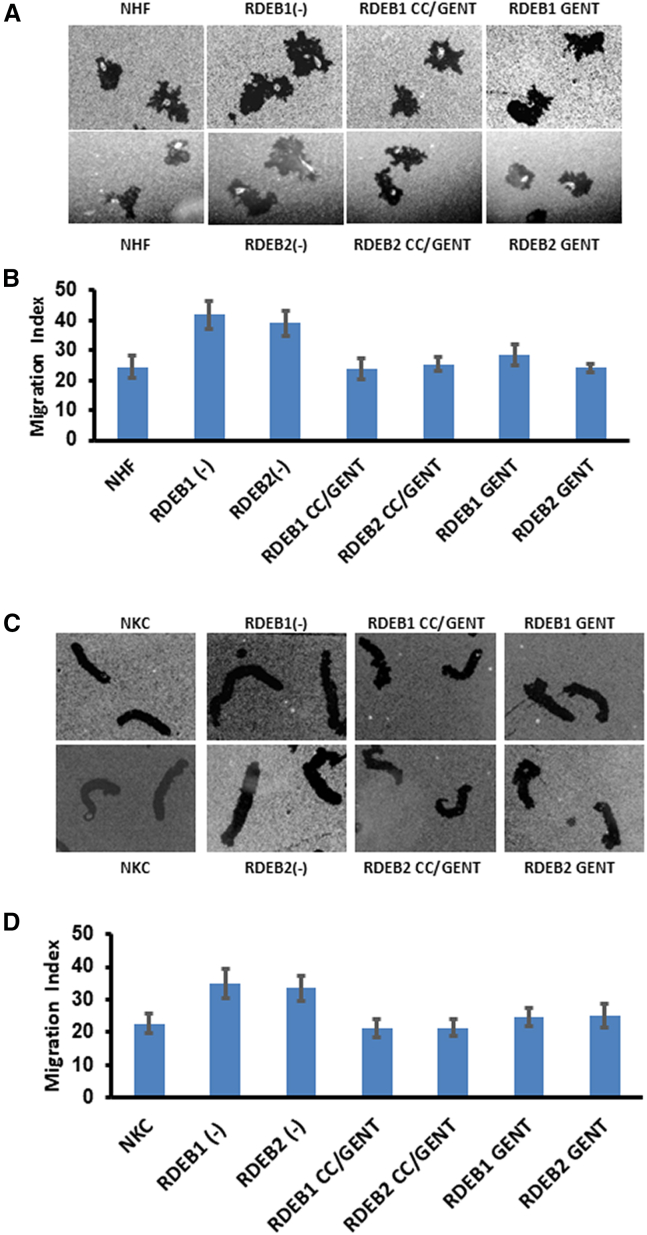
CC-90009 and gentamicin reversed RDEB fibroblast and keratinocyte hypermotility RDEB fibroblasts and keratinocytes, denoted as RDEB1 and RDEB2, were treated with 0.6 μM CC-90009 (CC) and 50 μg/mL gentamicin (GENT) in RDEB fibroblasts, or 120 nM CC-90009 and 50 μg/mL gentamicin in RDEB keratinocytes, or treated with high-dose gentamicin 200 μg/mL as indicated for 48 h and then subjected to a colloidal gold salt migration assay using collagen I as a matrix. The top panels are representative fields photographed at 40X under dark field optics (A and C). In the bottom are computer-generated migration indices for RDEB fibroblasts (B) and keratinocytes (D). The migration index is the percentage of the total field area occupied by migration tracks. Error bars, SE of three different experiments. Note that both untreated RDEB fibroblasts and keratinocytes showed hypermotility in comparison with normal human fibroblasts (NHFs) and normal human keratinocytes (NKCs). In contrast, treatment with either CC-90009/low-dose gentamicin or high-dose gentamicin corrected RDEB cell hypermotility.

While the primary goal of readthrough therapy is to achieve the suppression of PTCs, it is crucial that the resulting protein maintains functionality. The inhibition of PTCs involves a mispairing between the stop codon and near-cognate aminoacyl tRNA.^21^ This mechanism may yield a resulting protein with a substituted amino acid instead of the original one, potentially modifying the structure or function of the resulting protein product.^21^ In earlier studies, we established that RDEB fibroblasts and keratinocytes display heightened motility in comparison to NHFs and NKCs.^37^ Consequently, we aimed to investigate whether CC-90009 and gentamicin combination therapy could generate functional C7 to correct the hypermotility characteristic of RDEB fibroblasts and keratinocytes. RDEB1 and RDEB2 fibroblasts and keratinocytes, whether untreated or treated with CC-90009 and/or gentamicin, were subjected to a migration assay to evaluate their motility. Figure 4A presents microscopic fields of NHFs and RDEB1/RDEB2 fibroblasts under untreated conditions, treated with 0.2 μM CC-90009/low-dose gentamicin (50 μg/mL), or treated with high-dose gentamicin (200 μg/mL), while Figure 4C presents similar microscopic fields of NKCs and RDEB1/RDEB2 keratinocytes under untreated conditions, treated with 120 nM CC-90009/low-dose gentamicin (50 μg/mL), or treated with high-dose gentamicin (200 μg/mL). Cellular motility, quantified by the migration index (MI), was determined as the percentage of the microscopic field occupied by motility tracks.^37^ Prior to undergoing CC-90009 and gentamicin combination therapy, RDEB1 and RDEB2 fibroblasts exhibited increased motility, producing an MI of 41.94 and 39.12, respectively, compared to the MI of 24.47 observed for NHFs (Figure 4B). Similarly, RDEB1 and RDEB2 keratinocytes exhibited increased motility with MIs of 34.85 and 33.51, respectively, compared to the MI of 22.7 observed for NKCs (Figure 4D). After CC-90009/low-dose gentamicin treatment, RDEB1 and RDEB2 fibroblasts exhibited a reversal in hypermotility, displaying an MI of 23.82 and 25.47, respectively. RDEB1 and RDEB2 keratinocytes also exhibited a similar reduction in motility, with MIs of 21.29 and 21.41, respectively. Treatment with high-dose gentamicin alone also led to reduced motility in RDEB1 and RDEB2 cells; however, MI levels were comparable between high-dose gentamicin and CC-90009/low-dose gentamicin combination therapy. Taken together, these findings suggest that combination therapy with CC-90009 and gentamicin can generate functional C7 to correct the hypermotility characteristic of RDEB fibroblasts and keratinocytes.

### Combining CC-90009 and gentamicin reverses the hypermotility and poor substratum attachment observed in JEB keratinocytes

**Figure 5:**
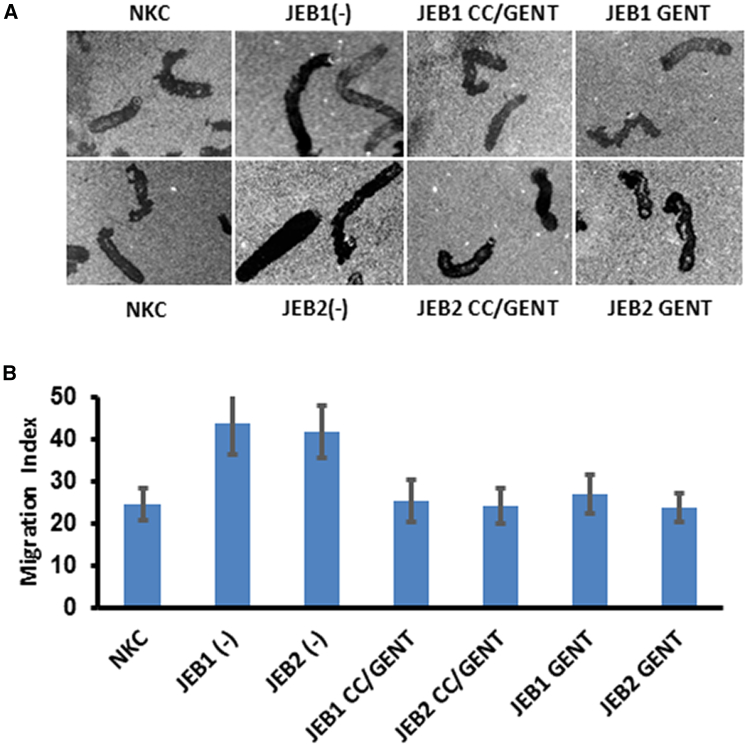
CC-90009 and gentamicin reversed JEB keratinocyte hypermotility JEB1/JEB2 keratinocytes were untreated, treated with CC-90009 (CC)/low-dose gentamicin (GENT), or treated with high-dose gentamicin (200 μg/mL) as indicated for 48 h and then subjected to a colloidal gold salt migration assay using collagen I as a matrix. JEB1 cells were treated with 100 nM CC-90009 and 50 μg/mL gentamicin, while JEB2 cells were treated with 100 nm CC-90009 and 12.5 μg/mL gentamicin. The top panels are representative fields photographed at 40X under dark-field optics (A). In the bottom are computer-generated migration indices for JEB1 and JEB2 keratinocytes (B). The migration index is the percentage of the total field area occupied by migration tracks. Error bars, SE of three different experiments. Note that both untreated JEB1/JEB2 keratinocytes showed hypermotility in comparison with normal human keratinocytes (NKCs). In contrast, treatment with either CC-90009/low-dose gentamicin or high-dose gentamicin corrected JEB cell hypermotility.

Given the abnormal cellular phenotypes observed in JEB cells, such as hypermotility and decreased cell adhesion,^38^ we sought to examine the functionality of laminin β3 produced by CC-90009 and gentamicin combination therapy. To achieve this, we conducted a keratinocyte migration assay and calculated MIs to evaluate the motility of both treated and untreated JEB keratinocytes. Figure 5A shows microscopic fields of NKCs and JEB1/JEB2 keratinocytes under untreated conditions, treated with CC-90009 andlow-dose gentamicin, or treated with high-dose gentamicin alone. Before undergoing CC-90009 and gentamicin combination therapy, JEB1 and JEB2 keratinocytes displayed increased motility, recording an MI of 43.7 and 41.77, respectively, in contrast to the MI of 24.62 observed for NKCs (Figure 5B). Treatment with high-dose gentamicin alone resulted in decreased motility in JEB1 and JEB2 cells, producing an MI of 23.79–26.85. However, combination therapy with both CC-90009 and low-dose gentamicin was comparable to high-dose gentamicin, resulting in a reversal of hypermotility with an MI of 25.39 and 24.05 in JEB1 and JEB2 cells, respectively.

**Figure 6:**
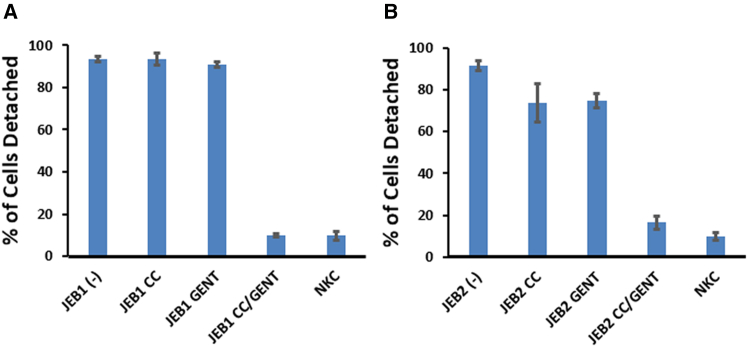
CC-90009/gentamicin reversed the poor substratum attachment of JEB cells JEB1 keratinocytes (A) were untreated or treated with 0.1 μM CC-90009 (CC), 50 μg/mL gentamicin (GENT) , or 0.1 μM CC-90009 and 50 μg/mL gentamicin for 48 h, while JEB2 keratinocytes (B) were untreated or treated with 0.1 μM CC-90009, 12.5 μg/mL gentamicin, or 0.1 μM CC-90009 and 12.5 μg/mL gentamicin for 48 h. All cells were simultaneously trypsinized, and the number of cells detached after 5 min was determined and expressed as a percent of the total number of cells for each patient’s cells. After 5 min, about 90% of JEB cells were detached, while normal human keratinocytes (NKCs) and CC-90009/gentamicin-treated JEB1 and JEB2 keratinocytes exhibited less than 20% detachment. Each value is the average of triplicates from three independent experiments.

We then assessed whether laminin β3, generated through CC-90009 and gentamicin combination therapy, could ameliorate the impaired cell-substratum adhesion in JEB cells. JEB1 and JEB2 cells underwent a well-established kinetic cell detachment assay under the following conditions: untreated, treated with CC-90009 alone, treated with low-dose gentamicin alone, or treated with CC-90009/low-dose gentamicin combination therapy. Subsequently, cells were detached from the substratum and quantified after a 5-min interval following the addition of trypsin. The results were subsequently presented as a percentage relative to the total cell count for each cell line and the concentration of CC-90009 and/or gentamicin treatment. Before treatment, JEB1 and JEB2 cells demonstrated weak cell-substratum adhesion, with more than 90% of JEB cells detaching within 5 min, in contrast to only 10% of NKCs (Figures 6A and 6B). Monotherapy with CC-90009 or gentamicin was ineffective for JEB1 cells and only minimally effective for JEB2 cells. However, treatment with the optimal concentrations of CC-90009 and gentamicin combination therapy improved their cell-matrix adhesion strength to a degree similar to that observed in NKCs. Monotherapy with either CC-90009 or low-dose gentamicin demonstrated limited effectiveness, but the combination of CC-90009 and low-dose gentamicin led to a reversal of the hypermotility and poor cell-substratum adhesion characteristic of JEB cells. This suggests a potential synergistic action between CC-90009 and gentamicin in correcting abnormal cellular phenotypes in JEB cells.

### CC-90009 and gentamicin combination therapy produces C7 and laminin β3 capable of localizing to the DEJ in RDEB and JEB skin equivalents

**Figure 7:**
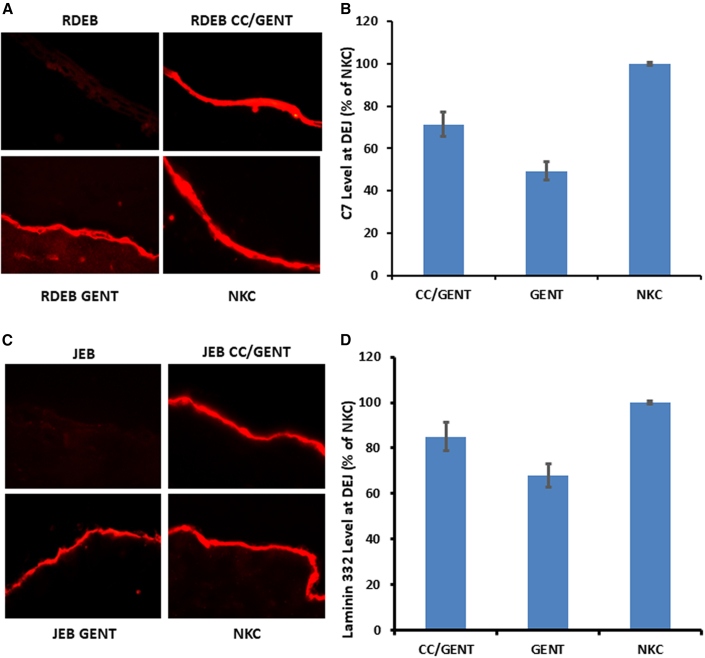
CC-90009/gentamicin-induced C7 or laminin 332 incorporated into the DEJ of *in vitro* skin equivalents Cryosections from 1-week skin equivalents (SEs) were subjected to immunofluorescent labeling using a polyclonal anti-C7 antibody (A) or anti-laminin 332 (β3) antibody (C). RDEB or JEB are SEs composed of RDEB2 keratinocytes or JEB2 keratinocytes combined with RDEB fibroblasts and normal fibroblasts. RDEB CC/GENT, JEB CC/GENT, RDEB/GENT, and JEB/GENT are SEs composed of RDEB fibroblasts and normal fibroblasts combined with RDEB or JEB keratinocytes treated with both CC-90009/low-dose gentamicin or gentamicin (200 μg/mL) before seeding and after plating onto dermal equivalents. RDEB CC/GENT were treated with 0.6 μM CC-90009 and 50 μg/mL gentamicin , while JEB CC/GENT were treated with 100 nM CC-90009 and 12.5 μg/mL gentamicin. NKCs are SEs composed of normal human fibroblasts combined with normal human keratinocytes. Intensity of C7 (B) or laminin 332 (D) at the DEJ of each specimen was measured by computer-assisted ImageJ software and compared to the intensity of C7 or laminin 332 in SEs derived from NKCs. Values represent the intensity of C7 or laminin 332 in the DEJ of the SEs expressed as a percentage of the average intensity obtained from NKCs (set as 100%). Data represent the mean ± SE.

After establishing that combination therapy with CC-90009 and gentamicin restores the production of full-length C7 and laminin β3 and corrects abnormal phenotypes in RDEB and JEB cells, our next goal was to investigate whether the newly produced C7 or laminin β3 incorporates into the DEJ. To explore this, *in vitro* three-dimensional organotypic skin equivalents (SEs) were created using RDEB2 and JEB2 keratinocytes, untreated, treated with CC-90009/low-dose gentamicin, or treated with high-dose gentamicin alone. Up to 2 weeks after establishing the SEs in culture, immunofluorescence staining was performed using a polyclonal antibody to C7 (A) or a polyclonal anti-laminin 332 (β) antibody recognizing the β3 chain (B). As anticipated, SEs derived from untreated RDEB or JEB keratinocytes exhibited no expression of C7 or laminin β3 at the DEJ (Figures 7A and 7C). In contrast, there was strong linear staining of laminin β3 at the DEJ in the SEs generated from keratinocytes treated with CC-90009/low-dose gentamicin, similar to SEs generated from normal cells. Quantitative analysis using ImageJ revealed that the amount of C7 deposited at the DEJ in SEs composed of CC-90009/low-dose gentamicin-treated RDEB keratinocytes was approximately 71.21% compared to the level of C7 produced in SEs composed of NKCs (Figure 7B). In addition, SEs composed of CC-90009/low-dose gentamicin-treated RDEB keratinocytes produced approximately 1.44 times the amount of C7 at the DEJ compared to SEs treated with high-dose gentamicin alone. For the SEs generated from JEB cells, the level of laminin 332 detected at the DEJ from CC-90009/low-dose gentamicin-treated cells was 84.92% of the level of laminin 332 produced from SEs composed of NKCs, approximately 1.25 times the amount of laminin 332 compared to SEs treated with high-dose gentamicin alone (Figure 7D). To confirm if this linear deposition was actually within the DEJ, the SE sections were co-labeled with a monoclonal anti-α6 integrin targeting the α6β4 integrin, an adhesion molecule located in basal keratinocytes that acts as the nucleating center for hemidesmosome formation in normal skin (Figure S6). Coimmunolabeling of SEs composed of JEB keratinocytes treated with CC-90009/low-dose gentamicin revealed strong linear staining of both α6 integrin and laminin 332 co-localized at the DEJ between the keratinocytes and the dermal equivalent. In contrast, SEs generated from untreated JEB kerationcytes displayed no staining, at the DEJ. Therefore, we conclude that CC-90009/gentamicin-induced C7 or laminin 332 is able to incorporate into its proper location at the DEJ *in vitro.*

## Discussion

In this study, we evaluated the effectiveness of CC-90009 and low-dose gentamicin combination therapy in inducing PTC readthrough in both RDEB and JEB. Our findings show that this combination increases the production of C7 in primary RDEB fibroblasts and keratinocytes, as well as laminin 332 in primary JEB keratinocytes, with increased potency over prolonged dosing. CC-90009 and low-dose gentamicin together proved more effective than high-dose gentamicin alone in promoting PTC readthrough, leading to higher levels of C7 and laminin 332 in RDEB and JEB cells, respectively. The induced C7 and laminin 332 were functional, as demonstrated by the reversal of hypermotility in RDEB and JEB cells and the correction of defective cell-substratum adhesion in JEB cells. Furthermore, C7 and laminin 332 produced by CC-90009/low-dose gentamicin-treated RDEB and JEB keratinocytes successfully incorporated into the DEJ in SE models.

In our study, CC-90009 monotherapy showed limited efficacy in RDEB fibroblasts and was largely ineffective in RDEB and JEB keratinocytes, suggesting cell type-dependent activity. By contrast, CC-90009 combined with gentamicin had strong synergistic effects, markedly increasing C7 and laminin 332 production beyond either agent alone, consistent with prior reports.^32^^,^^33^^,^^34^ This synergy likely reflects CC-90009-mediated eRF3a degradation and nonsense-mediated decay suppression together with gentamicin-induced ribosomal changes that enhance PTC readthrough.^29^ Supporting this, we observed eRF3a reduction after CC-90009 alone or combination treatment (Figure S7). Importantly, neither treatment altered normal protein expression or caused readthrough at native stop codons (Figure S8).

Research suggests that the readthrough potential of a PTC is affected by both the specific stop codon type (UGA>UAG>UAA) and the nucleotides directly following it (C>U>G>A). Specifically, the stop codon UGA, when followed by a C, exhibits the highest vulnerability to readthrough induced by aminoglycosides.^39^^,^^40^ In a prior investigation, our laboratory observed no discernible association between the readthrough efficacy of gentamicin and PTC mutations in 22 instances of RDEB caused by nonsense mutations. This finding remained consistent irrespective of the stop codon types, their contextual surroundings, and their proximity to exon-intron boundaries.^22^ While this study did not conduct a comprehensive analysis of the readthrough potential of individual mutations associated with RDEB or JEB, the consistent and strong response observed in both cell types across diverse mutations when treated with a combination of CC-90009 and gentamicin suggests the efficacy of the treatment independent of mutation sequence. However, it is important to recognize that the mutations we tested here are limited, and further experiments involving a wider range of mutations are necessary to determine whether CC-90009/gentamicin-induced PTC readthrough is mutation specific. Each cell type employed in the experiments carried a distinct mutation. Despite this diversity, the response to the combination therapy consistently resulted in robust protein production, including in JEB1 cells heterozygous for the clinically prevalent R635X mutation found in up to 84% of JEB patients with an altered *LAMB3* gene.^11^^,^^12^ Taken together, these results highlight the broad therapeutic potential of combing CC-90009 and gentamicin, supporting its application in promoting PTC readthrough in both RDEB and JEB, regardless of the specific mutation sequence.

Evidence indicates that restoring just 35% of normal C7 levels is sufficient to correct the RDEB phenotype and prevent blistering.^41^ In C7 knockout mice, epidermal-dermal adherence was maintained at this 35% threshold but was compromised when C7 levels fell below this critical threshold.^41^ Similarly, humans with one *COL7A1* null allele, resulting in 50% of the typical C7 and AFs, show no skin fragility, unlike homozygous relatives with RDEB.^41^^,^^42^ In this study, combination therapy with CC-90009 and low-dose gentamicin raised C7 above the critical 35% threshold, yielding functional protein that localized correctly to the DEJ and reduced hypermotility. These results demonstrate that this therapy produces sufficient, functional C7 to counteract RDEB pathology.

While approximately 35% of normal C7 is needed to correct RDEB, the threshold of laminin 332 required to correct JEB remains unclear. A JEB patient with spontaneous *LAMA3* readthrough improved clinically despite minimal laminin α3 secretion, suggesting lower amounts may suffice.^43^ In our prior work, gentamicin restored 12.8%–28.5% of normal laminin 332 in JEB keratinocytes.^23^ In this study, CC-90009 plus low-dose gentamicin was far more effective than either agent alone, increasing laminin β3 to 44.7% and 38.7% of normal levels in JEB1 and JEB2 cells. Compared with earlier protocols, our optimized regimen (100 nM CC-90009 and 12.5–50 μg/mL gentamicin) achieved maximal restoration. The generated laminin 332 reduced hypermotility, corrected adhesion defects, and localized correctly to the DEJ. Thus, CC-90009 plus low-dose gentamicin produces functional, properly localized laminin 332, fulfilling key objectives for JEB therapy.

Our research group has previously shown that RDEB fibroblasts and keratinocytes exhibit abnormal hypermotility, a phenotype that can be reversed upon restoration of functional C7.^37^ Similar findings of keratinocyte hypermotility have also been reported in JEB models.^44^ As a result, cell motility has become a commonly used functional assay in *in vitro* models of EB. Altered cell motility may impair coordinated re-epithelialization and contribute to a wound environment prone to fibrosis, characterized by elevated transforming growth factor β signaling and persistent inflammation.^45^^,^^46^^,^^47^ These factors are thought to play a role in downstream clinical complications such as mitten deformities and increased risk of aggressive cutaneous squamous cell carcinoma.^48^ Our findings that CC-90009/low-dose gentamicin combination therapy reduces the hypermotility of RDEB fibroblasts and keratinocytes, as well as JEB keratinocytes, suggest a potential therapeutic benefit—not only in restoring C7 and laminin 332 expression but also in modulating cell behavior relevant to wound repair and fibrotic disease progression.

Our previous work showed that gentamicin promotes PTC readthrough in RDEB and JEB *in vitro*, with clinical trials confirming improved wound closure and increased C7 or laminin 332 expression in patients.^22^^,^^23^^,^^24^^,^^49^^,^^50^^,^^51^ However, prolonged high-dose gentamicin raises concerns of ototoxicity and nephrotoxicity.^25^^,^^26^ In this study, combining CC-90009 with low-dose gentamicin enhanced C7 and laminin 332 production in RDEB fibroblasts, RDEB keratinocytes, and JEB keratinocytes more effectively than high-dose gentamicin alone. These findings suggest that CC-90009 plus low-dose gentamicin offers a safer strategy to boost readthrough, supported by pharmacokinetic data showing dose-dependent CC-90009 bioavailability.^29^

Several alternative treatment approaches have been proposed for both JEB and RDEB. For JEB, these include protein replacement therapy, bone marrow stem cell transplantation, and gene-corrected cultured keratinocyte autograft transplantation.^11^^,^^15^^,^^16^^,^^17^ In the case of RDEB, treatments under investigation include protein replacement therapy using topical, intradermal, or intravenous administrations of recombinant human C7 and cell therapy involving bone marrow stem cells, gene-corrected RDEB fibroblasts, or allogeneic ABCB5+ mesenchymal stromal cells.^52^^,^^53^^,^^54^^,^^55^^,^^56^^,^^57^^,^^58^ Recently, the FDA has approved two localized gene therapies Vyjuvek (beremagene geperpavec) and Zevaskyn (prademagene zamikerace) for RDEB and Filsuvez (birch triterpenes) for both RDEB and JEB.^7^^,^^8^^,^^9^ Despite progress, many of these treatments face challenges of variable efficacy, invasiveness, and high cost. There remains a critical need for effective therapies, particularly for JEB, given its early mortality. Large-scale data collections of skin and appendage phenotypes may help identify prognostic markers and optimize early, personalized treatment strategies for RDEB and JEB patients.^59^ Our findings suggest that combining CC-90009 with low-dose gentamicin offers a promising alternative for both RDEB and JEB: both drugs are intravenously administered, both avoid live cells or viral vectors (eliminating the need for immunosuppression), and gentamicin’s low cost and broad availability further support its potential.

Limitations of this study include a small sample size, a narrow range of PTC variants tested, and reliance on *in vitro* models, which may not fully replicate *in vivo* EB dynamics. As such, findings should be interpreted in the context of the study design, and further studies using additional PTC variants and *in vivo* models are warranted to validate these results.

In summary, this study shows that the combination of CC-90009 and low-dose gentamicin can induce PTC readthrough and restore functional C7 and laminin 332 in RDEB and JEB. This provides proof of concept for using CC-90009 and gentamicin to suppress PTCs and promote C7 and laminin 332 expression in patients with RDEB and JEB caused by nonsense mutations. Further evaluation of this combination therapy for treating nonsense mutations in RDEB and JEB is needed in clinical trials. Additionally, CC-90009/gentamicin-mediated PTC readthrough therapy could potentially be applied to other inherited skin disorders caused by nonsense mutations.

## Materials and methods

### Cell cultures

Primary dermal fibroblasts and epidermal keratinocytes from two RDEB patients, RDEB1 homozygous for R578X mutations and RDEB2 heterozygous for R163X and R1683X mutations, were previously established from the patients’ skin biopsies and cultured in DMEM/Ham’s F12 (1:1) supplemented with 10% fetal bovine serum for primary fibroblasts and in EpiLife media supplemented with human keratinocyte growth supplement (HKG) (Thermo Fisher Scientific, Waltham, MA) for RDEB keratinocytes.^60^ Primary JEB keratinocytes from two JEB patients, JEB1 heterozygous for R42X and R635X mutations and JEB2 heterozygous for C325X/c.629-12T>A mutations, were previously established from patient’s skin biopsies and cultured in EpiLife media supplemented with HKGs (Thermo Fisher Scientific, Waltham, MA).^61^ Primary human keratinocytes were purchased from Thermo Fisher Scientific (Waltham, MA). NHFs from neonatal foreskin were initiated into culture as described previously.^37^ Primary fibroblasts were passaged as they reached confluence, and all experiments were performed on cells between passages 4 and 6.

### Drug treatment and immunoblot analysis

In experiments aimed at inducing PTC readthrough, CC-90009 (Cayman Chemical Company, Ann Arbor, MI) and/or gentamicin (Sigma, St. Louis, MO) were administered to RDEB keratinocytes, RDEB fibroblasts, or JEB keratinocytes when they reached 70%–80% confluency. Cells were exposed to CC-90009 and/or gentamicin for 48 to 72 h.

To assess the cellular expression of C7 or laminin β3 protein, cellular extracts were prepared 48 to 72 h after incubation with the aforementioned drugs. The extracts were then subjected to 4%–12% SDS-PAGE (Bio-Rad, Hercules, CA), and the proteins were electrotransferred onto a nitrocellulose membrane. The presence of C7 was identified using polyclonal antibodies to the NC1 domain of C7, followed by a horseradish peroxidase-conjugated goat anti-rabbit immunoglobulin G (IgG) and enhanced chemiluminescence detection reagent (GE Healthcare, Buckinghamshire, UK). The presence of the laminin β3 monomer was detected with a monoclonal anti-laminin β3 antibody (Anti-Kalinin B1, clone 17, BD Biosciences, San Diego, CA).

To determine cellular expression of C7 after prolonged dosing with CC-90009 and gentamicin, RDEB fibroblasts and RDEB keratinocytes were plated at a density of 300,000 cells per well of 6-well plates. On the following day, media were replaced with fresh media and cells were untreated or treated with various concentrations of CC-90009 and gentamicin for up to 6 consecutive days. Cell lysis was performed on different days post-treatment, followed by immunoblot analysis using a polyclonal antibody to C7 and a monoclonal antibody to β-actin (loading control).

### Cell migration assay

Cell migration was evaluated as previously outlined.^62^ RDEB fibroblasts, RDEB keratinocytes, or JEB keratinocytes were seeded at a density of 300,000 cells per well in a 6-well plate. Following a 24-h incubation period, cells were either left untreated or subjected to treatment with CC-90009 and/or gentamicin for 24 h. The media and cell lines were then enriched with the specified compound doses. Following 48 h of treatment, the cells were sub-cultured and subjected to a cell migration assay. Colloidal gold salts were affixed to coverslips and coated with type I collagen (15 mg/mL). Fibroblast or keratinocyte cultures were suspended, placed on the coverslips, and allowed to migrate for 16–20 h. The cells were then fixed in 0.1% formaldehyde in phosphate-buffered saline and examined under dark-field optics using a video camera connected to a computer equipped with a frame grabber. The computer analyzed 15–20 non-overlapping fields in each experimental condition with NIH Image 1.6 and determined the percentage area of each field consumed by cell migration tracks to establish the MI.

### Cell detachment assay

To assess the extent of cellular adherence induced by CC-90009 and/or gentamicin combination therapy, a trypsin-based detachment assay was utilized.^23^ In brief, NKCs and primary JEB keratinocytes were plated on 12-well tissue culture plates at a density of 2 × 10^4^ cells per well. Twenty-four hours post-seeding, the medium was replaced with one containing CC-90009 and/or gentamicin. After 48 h, 250 μL of trypsin/EDTA was introduced to each well, and any detached cells were removed and quantified after 5 min. An additional 250 μL of trypsin/EDTA was added, and all remaining cells were allowed to detach and were subsequently counted. The percentages of cells detached were obtained, and the averages and standard deviations from three independent wells for each condition/cell line were calculated.

### Establishment of *in vitro* organotypic SEs and immunofluorescence microscopy

Establishment of an *in vitro* skin co-culture model was performed as previously described.^23^ In brief, a mixture of DMEM media (Corning, Discovery Labware, Bedford, MA) containing human RDEB fibroblasts (1 × 10^6^ cells/mL) was mixed with rat tail collagen 1 solution (2.5 mg collagen/mL) (Corning, Discovery Labware, Bedford, MA) and 10x DMEM (Corning, Discovery Labware, Bedford, MA). After neutralizing this solution with sodium bicarbonate, 1 mL portions were dispensed into each 12-well insert (ThinCert 12-well, 3 μM pore size, Greiner Bio-One, Thermo Fisher Scientific, Waltham, MA), and the gels were allowed to polymerize. The fibroblast-infused collagen gel was then submerged in serum-free DMEM and incubated for 24 h. Subsequently, the DMEM media were aspirated, and each dermal equivalent received a coating of 50 μL of 50 μg/mL fibronectin solution (Sigma, St. Louis, MO) in ultrapure water, followed by a 30-min incubation period. Meanwhile, RDEB and JEB keratinocytes, whether left untreated or subjected to pre-treatment with CC-90009 and/or gentamicin, were re-suspended in EpiLife media supplemented with HKGs + 5% fetal calf serum (FCS) (Thermo Fisher Scientific, Waltham, MA) at a concentration of 1 × 10^6^ cells/mL. Afterward, the gels were submerged in EpiLife media supplemented with HKGs + 5% FCS and cultured for up to 10 days with descending FCS concentrations (5%, 2%, and 0% FCS) every 2–3 days that a medium change occurred, with CC-90009 and/or gentamicin supplementation where appropriate. The cells in this solution were seeded onto the fibronectin over each gel and incubated for 45 min to facilitate cell adhesion. Between days 7 and 10 post-keratinocyte seeding, the SEs were harvested, soaked in PBS, and then placed on nitrocellulose strips. The nitrocellulose-bound SEs underwent a 90-min immersion in a 50% sucrose solution before being slow-frozen on a metal plate over dry ice. Frozen SEs were mounted in optical cutting temperature (OCT) and frozen. Sections measuring five micrometers in thickness were cut from the OCT-embedded SEs using a cryostat. These sections were then fixed for 5 min in cold acetone and air-dried. Immunolabeling of the SEs was then conducted using standard immunofluorescence methods, as previously described.^23^ SE sections were labeled with either a polyclonal antibody against C7 or a polyclonal anti-laminin 332(β) chain antibody, followed by a CY3-conjugated goat anti-rabbit IgG (1:1,000). Representative images of the stained sections were captured using a Zeiss Axioplan fluorescence microscope equipped with a Zeiss Axiocam MRM digital camera system. All photographs were taken using the same camera at identical exposure times. The mean fluorescence intensity at the DEJ was determined for each sample using ImageJ (Rasband WS, NIH, Bethesda MD; http://rsb.info.nih.gov/ij/), following the previously outlined methodology. The mean fluorescence intensity at the DEJ was determined for each sample using ImageJ (Rasband WS, NIH, Bethesda MD; http://rsb.info.nih.gov/ij/), following the previously outlined methodology.^50^

### Cell viability

In the cytotoxicity assay for CC-90009 and/or gentamicin, RDEB fibroblasts were plated at a density of 20,000 cells per well of a 96-well plate. RDEB keratinocytes were plated at a density of 25,000 cells per well. JEB keratinocytes were plated at a density of 25,000 cells per well of a type I collagen-coated 96-well plate (required for cell attachment). At 24 h, cells were untreated or treated with escalating doses of CC-90009 and/or gentamicin for 24 h. After another 24 h, media and compounds were replaced with fresh media supplemented with indicated doses of compounds. Plates were allowed to incubate for 48 h. A freshly prepared solution of 4 mg 2,3-bis-(2-methoxy-4-nitro-5-sulfophenyl)-5[(phenylamino)carbonyl]- 2H-tetrazolium hydroxide (XTT, Visalia, CA) in 4 mL of culture medium was mixed with 10 μL of phenazine methosulfate (PMS; Sigma, St. Louis, MO) solution (3 mg of PMS in 1 mL of PBS), and 25 μL of the combined XTT/PMS solution was directly added to each 100 μL cell culture.^14^ Cultures were incubated for 4 h at 37°C, and absorbance was read at 570 and 600 nm.

## Data and code availability

The authors confirm that the data supporting the findings of this study are available within the article and its supplemental information.

## Acknowledgments

This work was supported by funding from the Epidermolysis Bullosa Research Partnership.

## Author contributions

Conceptualization: M.C. and B.L.; funding acquisition: M.C.; investigation: K.L.M., B.L., R.H., Y.H., K.Z., and M.C.; project administration and supervision: M.C.; validation: K.L.M., B.L., R.H., Y.H., K.Z., and M.C.; writing – original draft preparation: K.L.M., B.L., and M.C.; writing – review and editing: K.L.M., B.L., R.H., Y.H., K.Z., and M.C.

## Declaration of interests

The authors declare no competing interests.

## Supplemental information

Document S1. Figures S1–S8

Document S2. Article plus supplemental information
