# Supplementary material for: Novel eRF3a degrader enhances gentamicin-induced premature termination codon readthrough in epidermolysis bullosa
Source: Mol Ther Nucleic Acids. 2025 Oct 13;36(4):102741. doi: 10.1016/j.omtn.2025.102741 (PMC12594917; doi:10.1016/j.omtn.2025.102741)
Supplement: Document S1. Figures S1–S8 [file mmc1.pdf]

OMTN, Volume *36*

Supplemental information

Novel eRF3a degrader enhances gentamicin-induced premature termination codon readthrough in epidermolysis bullosa

Kathleen L. Miao, Brandon Levian, Yingping Hou, Ryan Huynh, Kate Zheng, and Mei Chen

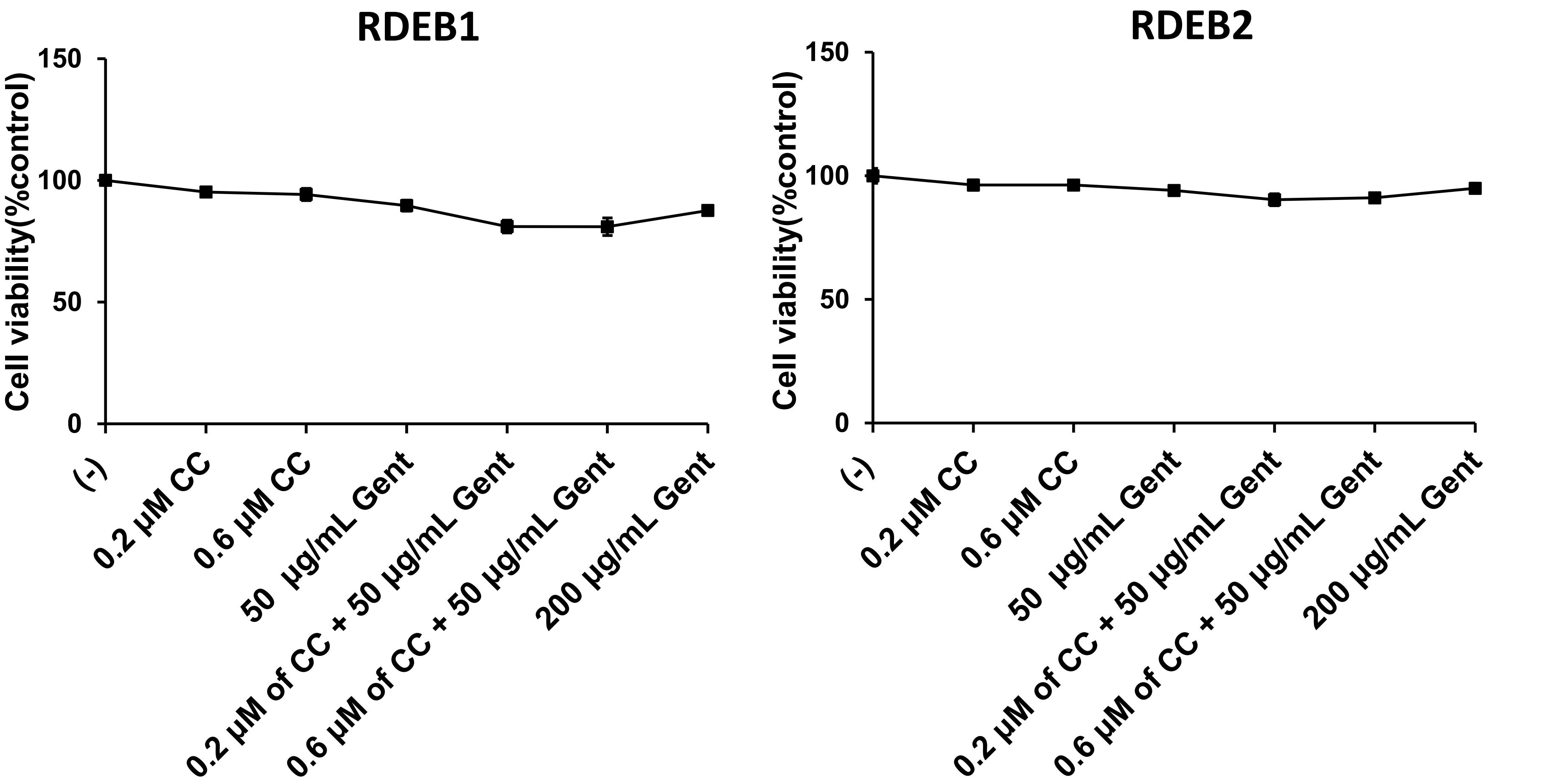

Figure S1. Viability Assay (XTT) of RDEB fibroblasts treated with CC-90009/gentamicin.
The primary RDEB fibroblasts (RDEB1 and RDEB2) were seeded onto 96 well plates in quadruplicate at a density of 10,000 cells per well. At 24 hours after seeding, fresh medium was added containing CC-90009 and/or gentamicin. After another 24 hours, media and compounds were then replaced with fresh media supplemented with indicated doses of CC-90009 and/or gentamicin . At 48 hours after treatment, XTT solution was added to each well. After 4 hours of incubation at 37°C, plates were read at a 570 nm and 600 nm wavelengths. Data represent the mean ± SD of quadruplicate determinations in one representative experiment. Similar results were obtained in two other independent experiments.

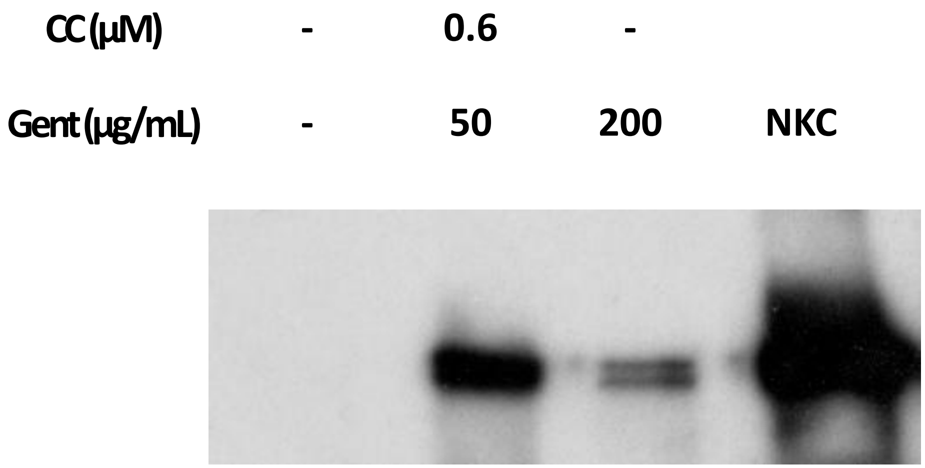

Figure S2. CC-90009 and gentamicin-induced full-length C7 secreted into cell media.
RDEB2 keratinocytes were incubated with growth media in the absence or presence of CC-90009/gentamicin or gentamicin alone for 48 hours. The conditioned medium was harvested at 72 hours after drug treatment and concentrated via Amicon Ultra-15. Equal amounts of each sample were separated on a 4–12% SDS-PAGE and analyzed by immunoblotting with a rabbit polyclonal antibody against the NC1 domain of C7 antibody. Please note that CC-90009/gentamicin or gentamicin-induced C7 was able to secrete into the media.

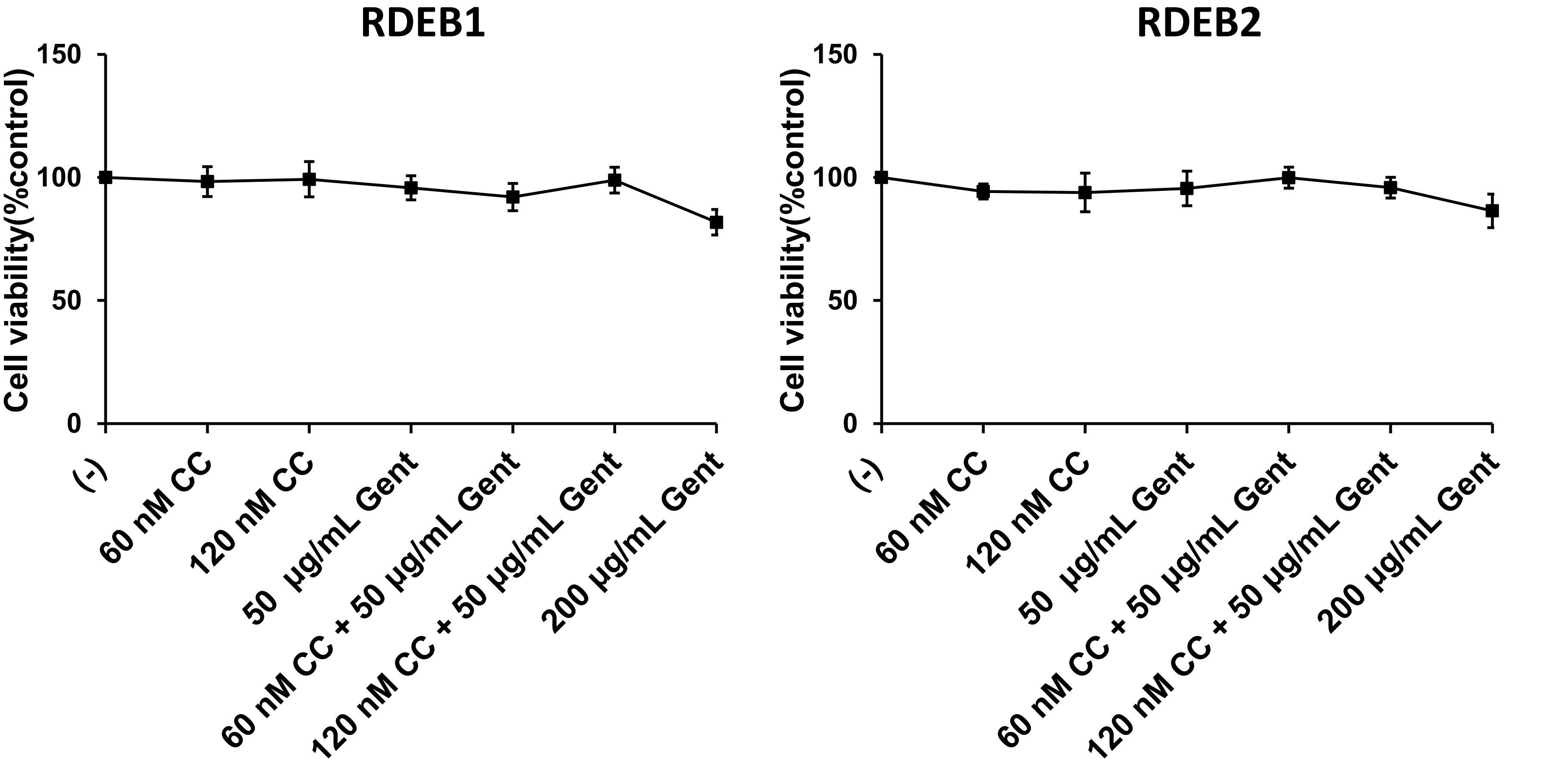

Figure S3. Viability Assay (XTT) of RDEB keratinocytes treated with CC-90009/gentamicin.
The primary RDEB keratinocytes (RDEB1 and RDEB2) were seeded onto 96 well plates in quadruplicate at a density of 15,000 cells per well. At 24 hours after seeding, fresh medium was added containing CC-90009 and/or gentamicin . After another 24 hours, media and compounds were then replaced with fresh media supplemented with indicated doses of CC-90009 and/or gentamicin. At 48 hours after treatment, XTT solution was added to each well. After 4 hours of incubation at 37°C, plates were read at a 570 nm and 600 nm wavelengths. Data represent the mean ± SD of quadruplicate determinations in one representative experiment. Similar results were obtained in two other independent experiments.

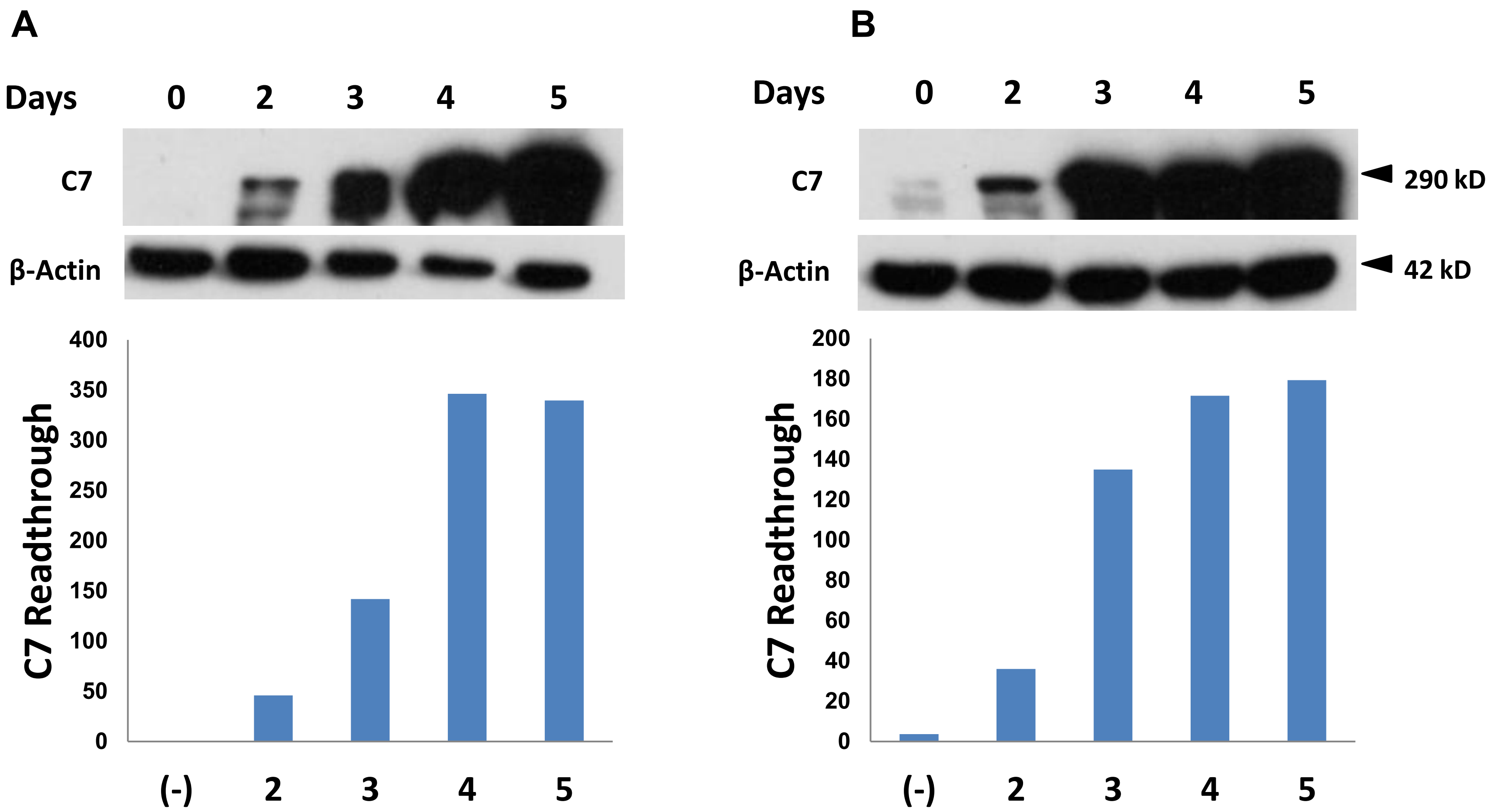

Figure S4. CC-90009 and gentamicin-induced production of full-length C7 increased with continued dosing.
RDEB1 fibroblasts (A) and RDEB1 keratinocytes (B) were incubated with growth media in the absence of CC-90009/gentamicin or were given consecutive daily treatments of CC-90009/gentamicin for up to five days. RDEB1 fibroblasts were treated with 0.6 μM CC-90009 and 50 μg/mL gentamicin, while RDEB1 keratinocytes were treated with 120 nM CC-90009 and 50 μg/mL gentamicin. Cell lysates were prepared and then subjected to 4-12% SDS-PAGE, followed by immunoblot analysis with a rabbit polyclonal antibody to the NC1 domain of C7 or anti-b-actin (loading control) antibody. The results are displayed as a fraction of the level of C7 obtained from the 5th dose of CC-90009/gentamicin. Please note that CC-90009/gentamicin-induced full-length C7 expression increased with daily treatment.

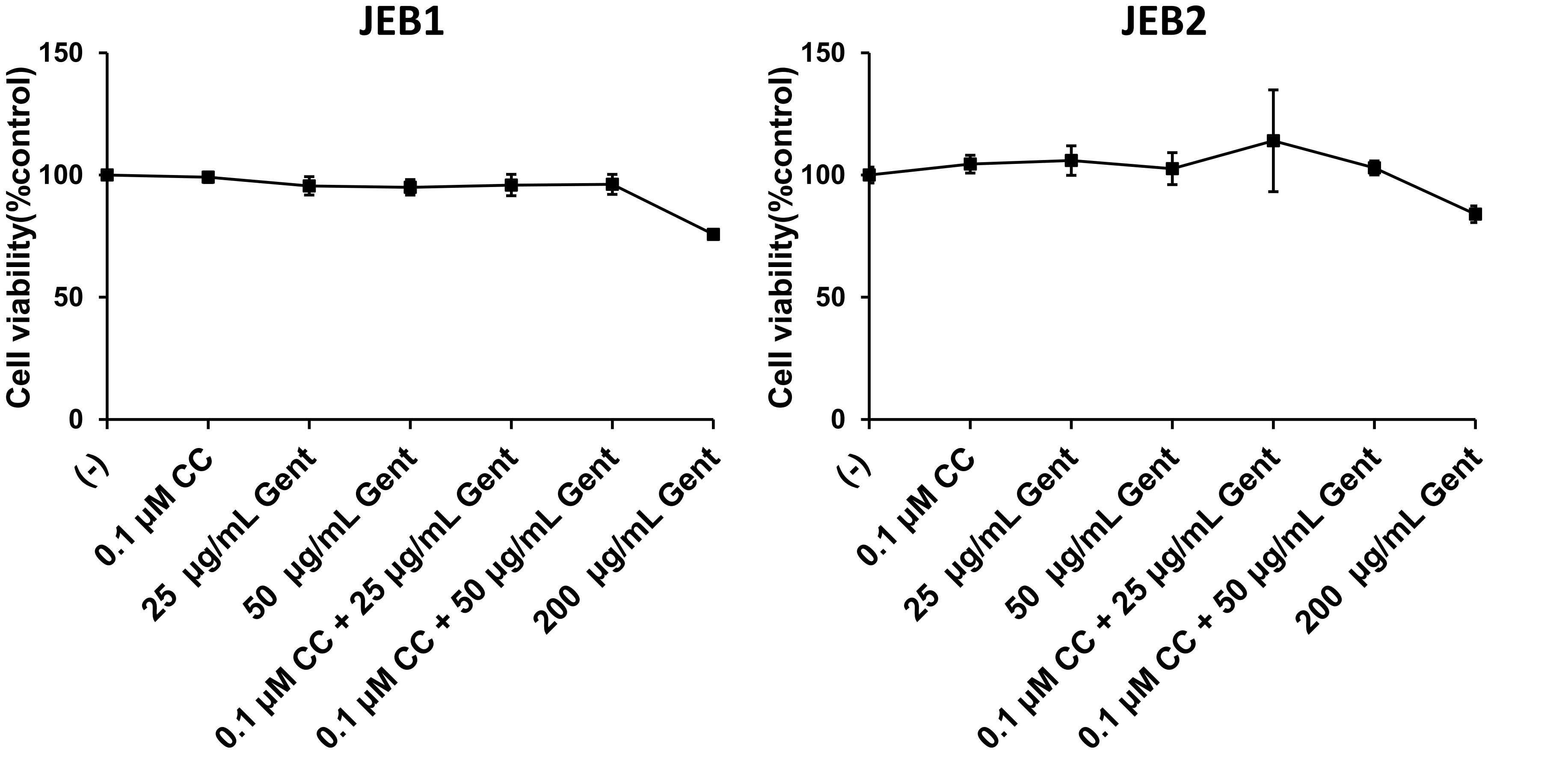

Figure S5. Viability Assay (XTT) of JEB keratinocytes treated with CC-90009/gentamicin.
The primary JEB keratinocytes (JEB1 and JEB2) were seeded onto 96 well plates coated with collagen 1 in quadruplicate at a density of 15,000 cells per well. At 24 hours after seeding, fresh medium was added containing CC-90009 and/or gentamicin. After another 24 hours, media and compounds were then replaced with fresh media supplemented with indicated doses of CC-90009 and/or gentamicin. At 48 hours after treatment, XTT solution was added to each well. After 4 hours of incubation at 37°C, plates were read at a 570 nm and 600 nm wavelengths. Data represent the mean ± SD of quadruplicate determinations in one representative experiment. Similar results were obtained in two other independent experiments.

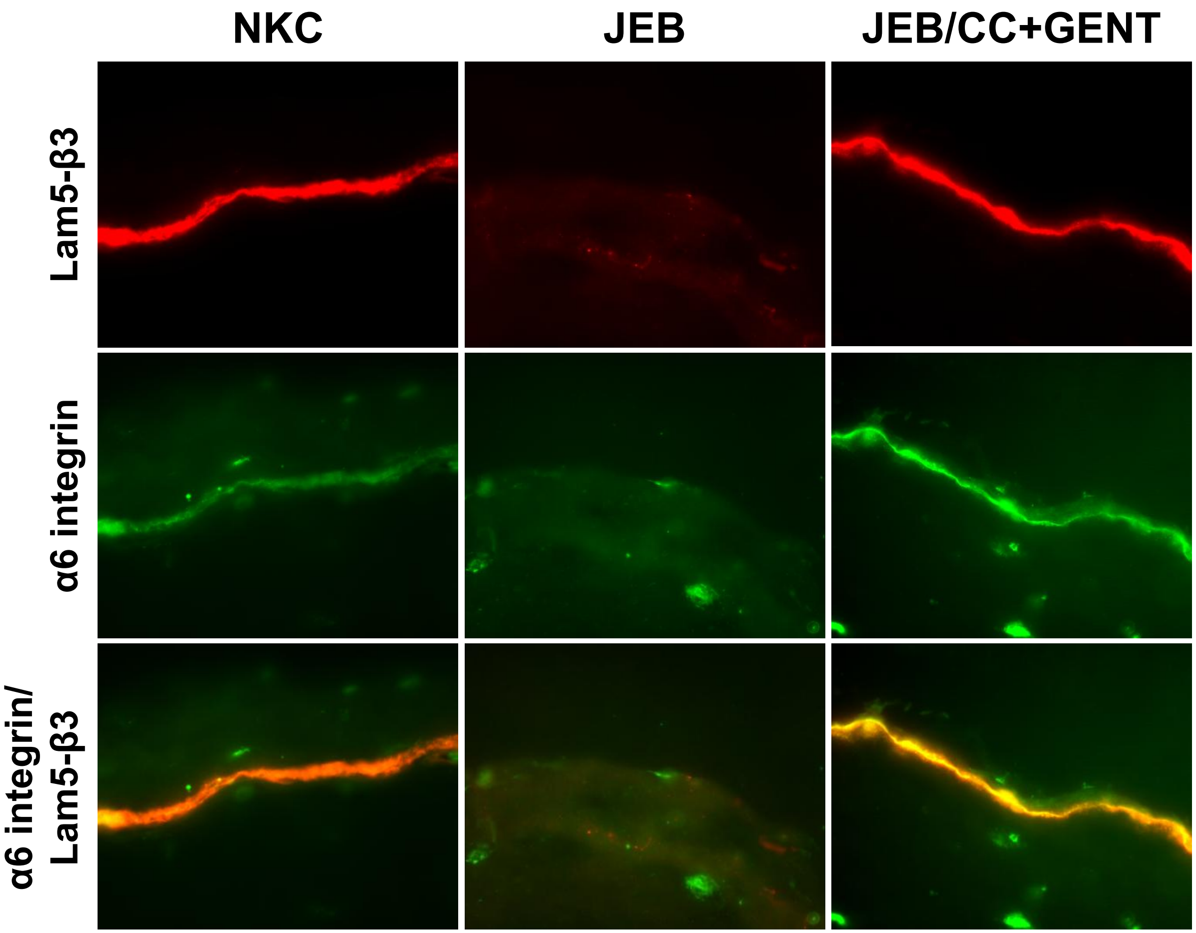

Figure S6. CC-90009/gentamicin-induced laminin 332 incorporated into the DEJ of in vitro skin equivalents (SEs).
Cryosections from 1 week old SEs were subjected to immunofluorescent labeling using a polyclonal anti-laminin 332 antibody [Lam-332(β3); row 1], then co-labeled with antibodies to the α6 integrin chain (α6 integrin, row 2). From left to right, the columns represent SEs established from normal human keratinocytes (NKC), untreated JEB (JEB) and JEB treated with CC/gentamicin (JEB/CC+GENT). Results are representative images from triplicate SEs. Note that the antibodies to the α6 integrin colocalize with the labeling of the Lam-332(β3) antibody.

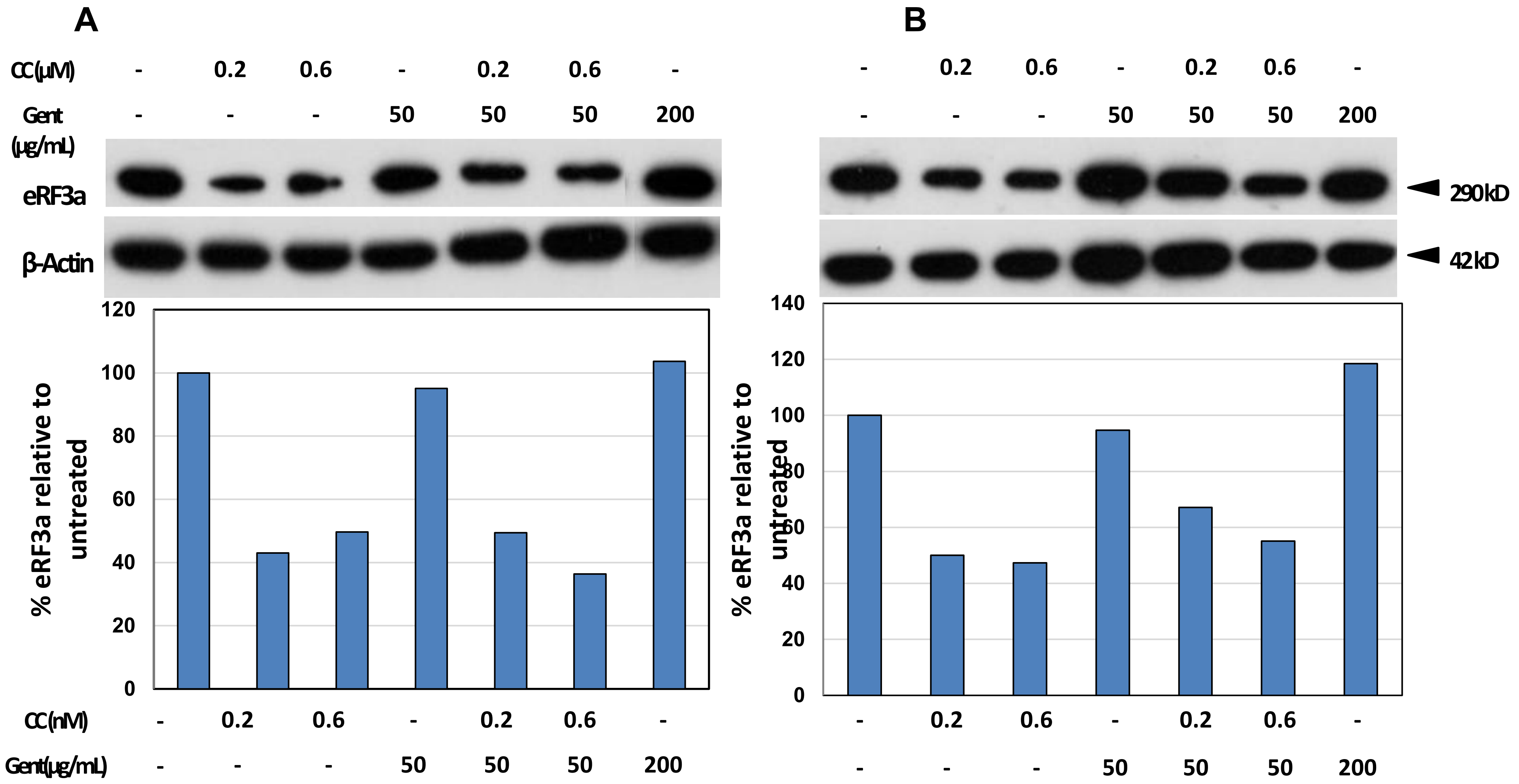

Figure S7. CC-90009 alone and in combination with gentamicin decreased eRF3a production in RDEB fibroblasts.
RDEB fibroblasts, denoted as RDEB1 (A) and RDEB2 (B) were treated with increasing concentrations of CC-90009 (CC) and gentamicin (Gent) as indicated, for 48 hours. Cell lysates were prepared and then subjected to 4-12% SDS-PAGE, followed by immunoblot analysis with an anti-eRF3a antibody or anti-b-actin (loading control) antibody. ImageJ analysis of eRF3a expression normalized with β-actin is shown below the respective blots. The results are displayed as compared to untreated RDEB fibroblasts.

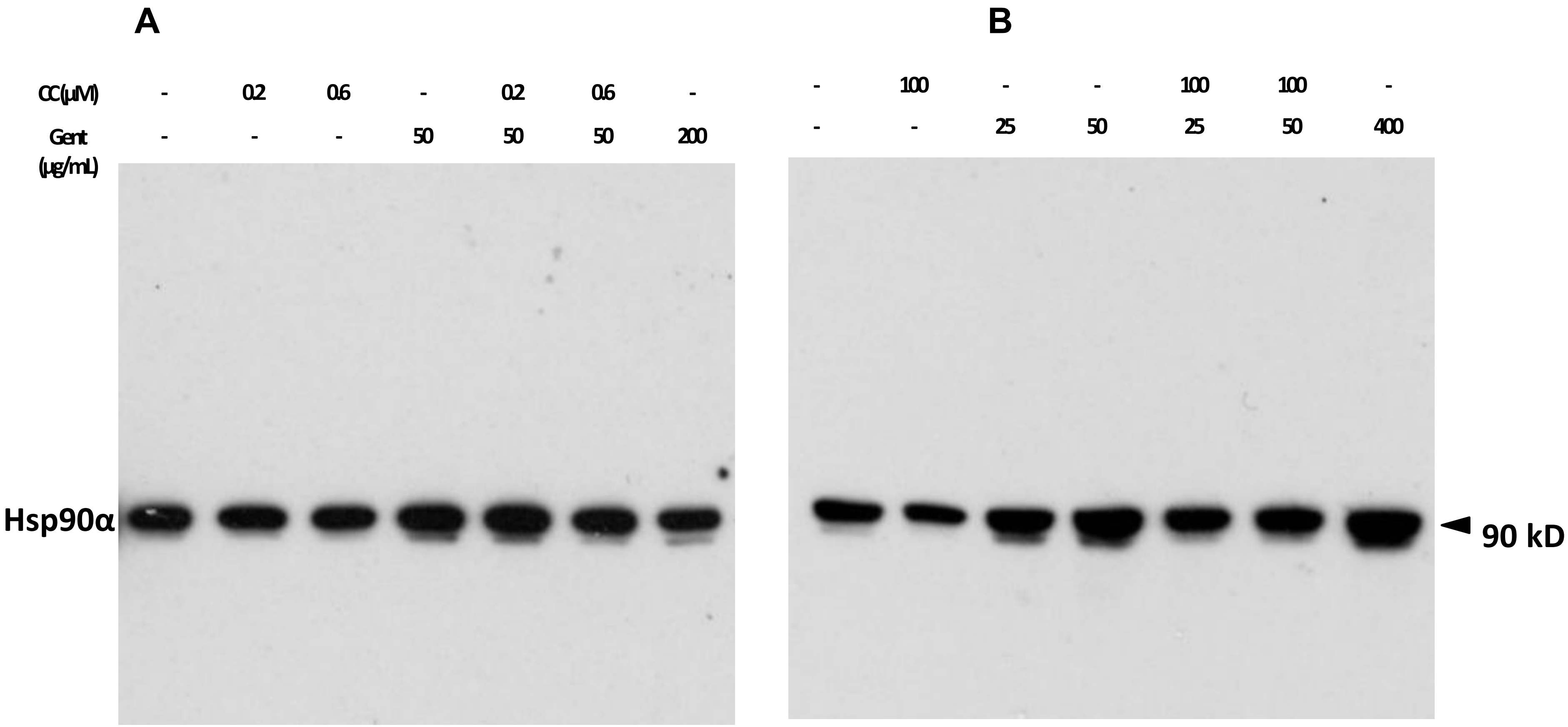

Figure S8. CC-90009, gentamicin, or CC-90009/gentamicin did not readthrough the normal stop codon.
RDEB fibroblasts (A) and JEB keratinocytes (B) were treated with increasing concentrations of CC-90009 (CC) and gentamicin (Gent) as indicated, for 48 hours. Cell lysates were prepared and then subjected to 4-12% SDS-PAGE, followed by immunoblot analysis with an anti-Hsp90 alpha antibody. No additional bands above 90 kD were noted in immunoblot analysis, indicating no additional readthrough activity past normal stop codons with CC-90009 and/or gentamicin treatment.
